# Automated quantification of the phagocytosis of *Aspergillus fumigatus* conidia by a novel image analysis algorithm

**DOI:** 10.3389/fmicb.2015.00549

**Published:** 2015-06-09

**Authors:** Kaswara Kraibooj, Hanno Schoeler, Carl-Magnus Svensson, Axel A. Brakhage, Marc Thilo Figge

**Affiliations:** ^1^Applied Systems Biology, Leibniz Institute for Natural Product Research and Infection Biology – Hans Knöll InstituteJena, Germany; ^2^Faculty of Biology and Pharmacy, Friedrich Schiller University JenaJena, Germany; ^3^Department of Molecular and Applied Microbiology, Leibniz Institute for Natural Product Research and Infection Biology – Hans Knöll InstituteJena, Germany

**Keywords:** *Aspergillus fumigatus*, alveolar macrophages, host-pathogen interaction, phagocytosis assay, automated image analysis

## Abstract

Studying the pathobiology of the fungus *Aspergillus fumigatus* has gained a lot of attention in recent years. This is due to the fact that this fungus is a human pathogen that can cause severe diseases, like invasive pulmonary aspergillosis in immunocompromised patients. Because alveolar macrophages belong to the first line of defense against the fungus, here, we conduct an image-based study on the host-pathogen interaction between murine alveolar macrophages and *A. fumigatus*. This is achieved by an automated image analysis approach that uses a combination of thresholding, watershed segmentation and feature-based object classification. In contrast to previous approaches, our algorithm allows for the segmentation of individual macrophages in the images and this enables us to compute the distribution of phagocytosed and macrophage-adherent conidia over all macrophages. The novel automated image-based analysis provides access to all cell-cell interactions in the assay and thereby represents a framework that enables comprehensive computation of diverse characteristic parameters and comparative investigation for different strains. We here apply automated image analysis to confocal laser scanning microscopy images of the two wild-type strains ATCC 46645 and CEA10 of *A. fumigatus* and investigate the ability of macrophages to phagocytose the respective conidia. It is found that the CEA10 strain triggers a stronger response of the macrophages as revealed by a higher phagocytosis ratio and a larger portion of the macrophages being active in the phagocytosis process.

## Introduction

The use of sophisticated microscopy techniques and the ease of producing and storing large amount of image data has in the last decade led to an increasing need for automated image analysis tools that reveal and quantify biological processes on a systems biology level (Rittscher, 2010). We will here present an automated image analysis algorithm that is able to fully evaluate the data from a phagocytosis assay between murine alveolar macrophages and the fungus *Aspergillus fumigatus*. This is a biologically relevant experiment as the ubiquitous saprophytic mold *A. fumigatus* is the most prevalent airborne fungal pathogen (Brakhage et al., 1999; Brakhage and Langfelder, 2002; O'Gorman et al., 2008). During its asexual reproduction cycle the fungus produces conidia that are inhaled by humans at a rate of hundreds to thousands per day, without any consequences for healthy humans (Latgé, 1999). In immunocompromised subjects, by contrast, *A. fumigatus* can cause invasive pulmonary aspergillosis (IPA) which has mortality rates in the order of 30–95% (Brakhage, 2005; Dagenais and Keller, 2009). From the host side, macrophage phagocytosis is part of the early response of the innate immune system and the igniting process of the adaptive immune system at a later stage (Aderem and Underhill, 1999). Therefore, *in vitro* phagocytosis assays where *A. fumigatus* conidia are confronted with mammalian macrophage cells are a suitable experiment to examine the interaction between pathogen and host, thereby gaining deeper insight into mechanisms of pathogenicity and phagocytosis.

The evaluation of phagocytosis assays based on images is often carried out by visual inspection and is therefore time consuming, subjective and expensive, accentuating the need for a more efficient analysis method (Zhou and Wong, 2006). The automated image analysis performed in this work was realized within the *Definiens Developer XD* framework (Schönmeyer et al., 2011) that allows creating customized algorithms tailored for the life sciences (Carpenter et al., 2006) and facilitates automated analysis of big sets of image data by batch processing. Additionally, this platform enables to conveniently combine predefined features of image objects by mathematical and logical combinations. Studying biological phenomena often requires analyzing microscopy images with a high degree of variation in object features, both across the images and even across objects in the same image. A clear example in the present context is the variation in shape of clusters formed by different numbers of conidia whose relative positions in the clusters are different for virtually all clusters. Another example is the change of the mean intensity of objects depending on how deep a cell lies in the experimental well. Validation of our algorithm ensures its applicability to a wide spectrum of image data required for high-throughput screening of mutants in comparative studies.

Here, we performed confocal laser scanning microscopy (CLSM) experiments to study two clinical isolates of *A. fumigatus*: ATCC 46645 (American Type Culture Collection, Manassas, VA) and CEA10. These two strains, among others, were examined for virulence in an embryonated egg model and CEA10 displayed an increased virulence compared with ATCC (Jacobsen et al., 2010). It should be noted that in this study older embryos had an increased survival chance and this was hypothesized to be caused by a more mature immune system. By performing the confrontation assay carried out here, we wanted to elucidate the infection process of the two strains and shed light on the mechanisms of CEA10's virulence, especially its interaction with a mature immune system. In our experiment we compared the phagocytosis ratio, macrophage-adherence and aggregation of the two strains.

In the microscopy experiments performed for the present study, different fluorescent dyes for macrophages and conidia were used and, in addition, the technique of differential staining was applied to distinguish phagocytosed from non-phagocytosed conidia (Thywißen et al., 2011). In the standard red-blue-green (RGB) formulation of a color image, each color layer displays a specific class of cells, i.e., all macrophages in the red layer, all conidia in the green layer and all non-phagocytosed conidia in the blue layer. Hence, the staining protocol enabled us to conveniently work on single layers for object segmentation and to ultimately combine layers in the classification of objects and in the analysis of their spatial colocalizations. While some progress has been made in previous developments of algorithms for the automated image analysis of this type of experiments (Mech et al., 2011, 2014; Kraibooj et al., 2014; Schäfer et al., 2014), we are here presenting a novel algorithm that differs from previous approaches with regard to the crucial step of segmenting all different types of cells in the assay. This was not achieved in previous approaches regarding the segmentation of macrophages, which is complicated by the occasionally interrupted staining of their cell surface. Since reliable segmentation of all cells in the assay is a necessary prerequisite for its comprehensive quantification, we could go beyond previous work and computed various biological quantities, such as the phagocytic index (Sano et al., 2003) and other measures involving the quantification of macrophages, which was not possible in previous studies.

## Materials and methods

### *A. fumigatus* strains and growth condition

Cultivation of the *A. fumigatus* wild-type ATCC 46645 and CEA10 was performed on *Aspergillus* minimal medium (AMM) agar plates with 1% (w/v) glucose at 37°C for 5 days. AMM consisted of 70 mM NaNO_3_, 11.2 mM KH_2_PO_4_, 7 mM KCl, 2 mM MgSO_4_ and 1 μl/ml trace element solution (pH 6.5). The trace element solution consisted of 1 g FeSO_4__*_ 7 H_2_O, 8.8 g ZnSO_4__*_ 7 H_2_O, 0.4 g CuSO_4__*_ 5 H_2_O, 0.15 g MnSO_4__*_ H_2_O, 0.1 g NaB_4_O_7__*_ 10 H_2_O, 0.05 g (NH_4_)_6_Mo_7_O_24__*_ 4 H_2_O, and double-distilled water (ddH_2_O) to 1000 ml (Brakhage and Van den Brulle, 1995; Maerker et al., 2005). Conidia were harvested in sterile, double-distilled water.

### Phagocytosis assays and cell staining

For the phagocytosis assays murine alveolar macrophages (ATCC CRL-2019™) were cultivated in RPMI1640 medium supplemented with 10% (v/v) FCS (Thermo Fisher Scientific, Dreieich, Germany), 1% (w/v) sodium bicarbonate (Lonza, Köln, Germany) and 0.05 mM beta-mercaptoethanol (Life Technologies, Darmstadt, Germany). The cells were seeded on glass cover slips in Nunc 24 well plates (Thermo Scientific) at a density of 3 × 10^5^ cells per well and allowed to grow adherently overnight. The conidia were stained with Fluorescein isothiocyanate (FITC, Sigma, Taufkirchen, Germany) for 30 min at 37°C while shaking. After washing them 3 times with PBS, 0.01% (v/v) Tween20 (AppliChem, Darmstadt, Germany) conidia concentration was determined using a CASY cell counter model TT (Roche-Innovatis, Penzberg, Germany). Conidia were added to the macrophages at a multiplicity of infection (MOI) of 7. Synchronization of the experiment was realized by centrifugation for 5 min at 100 g and 37°C. To initiate the experiment the co-incubation was shifted to a humidified CO_2_ incubator for 1 h at 37°C. The cells were fixed for 15 min at room temperature by adding 16% (v/v) paraformaldehyde (Electron Microscopy Sciences, München, Germany) directly to the medium to a concentration of 3.7% (v/v). Wells were washed two times with PBS followed by the step of differential staining, i.e., non-phagocytosed conidia were stained with 0.1 mg/ml calcofluor white (Sigma) for 30 min at room temperature. The cells were washed again twice with PBS. Prior to antibody labeling, binding sites were blocked with PBS, 3% (w/v) BSA Fraktion V (AppliChem) for 30 min. Next, macrophages were labeled with a monoclonal rat anti-CD9 antibody (1:200; Santa Cruz Biotechnology, Heidelberg, Germany) over night at 4°C and an Alexa Fluor® 647 Goat Anti-Rat IgG antibody (1:200; Life Technologies) for 1.5 h at room temperature.

### Imaging

**Figure 1:**
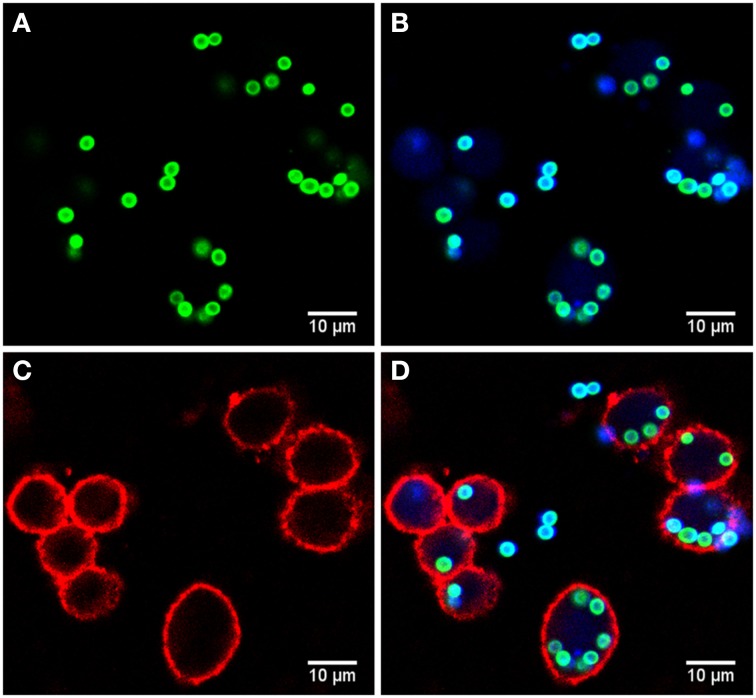
**Example of image data from the phagocytosis assay. (A)** Green layer (FITC staining) with all conidia. **(B)** Blue layer (Calcoflour white staining) overlaid with the FITC layer revealing the difference between phagocytosed and non-phagocytoced conidia as only the latter were stained with Calcoflour white (differential staining). **(C)** Red layer (anti-CD9 antibody) with macrophages and **(D)** overlay of all layers.

Microscopy images were taken by a Zeiss LSM 780 Live confocal laser scanning microscope with a 20x Zeiss plan-apochromat dry objective (0.8 NA). The resulting images are 8-bit RGB color images with 1024 × 1024 pixels and a pixel distance of 0.2 μm. The total number of images for each strain is 60, equally divided into two biological replicates (i.e., 30 images each) with two technical replicates per biological replicate (i.e., 15 images each). These image data are publicly available at http://www.leibniz-hki.de/en/asb-downloads.html. Based on the differential staining, all macrophages appeared in the red layer, all conidia in the green layer and all non-phagocytosed conidia were visible in the blue layer (see Figure 1). The separation of objects into different layers allowed for more effective segmentation and classification of the different image objects.

### Automated image analysis

**Figure 2:**
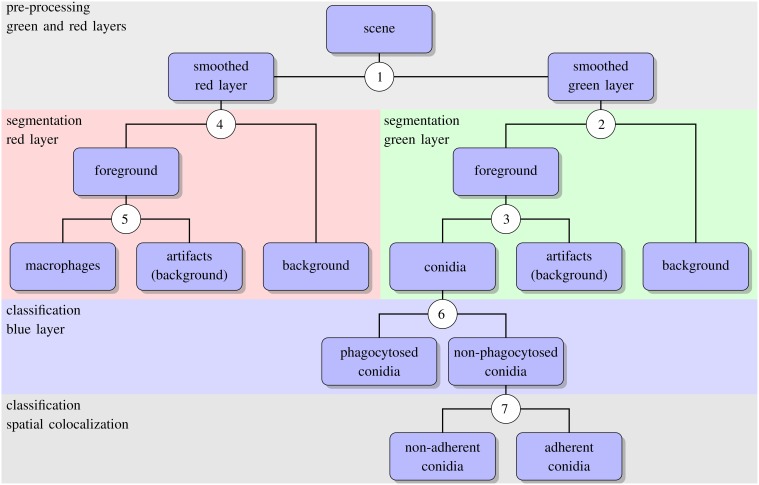
**Schematic representation of the automated image analysis algorithm with boxes showing input and output of image analysis processes**. Circled numbers indicate split points where the different processes were performed, e.g., thresholding or object classification. For the details of each split point we refer the reader to the text.

The algorithm for automated image analysis was developed using the software *Definiens Developer XD* and was executed by the *Grid XD Server* (Definiens AG, Munich, Germany). The server ran on one core of a SUN Fire X4600 Server M2 (8 CPUs with 4 cores each, 2.3 GHz AMD Opteron, 64 GB memory). Image processing consisted of three subsequent steps—preprocessing, segmentation and classification—and a schematic overview of the algorithm is presented in Figure 2. The rule set of the algorithm is provided as Supplementary Material (see Supplementary Data 1) and the code is available by the authors upon request.

#### Preprocessing

**Figure 3:**
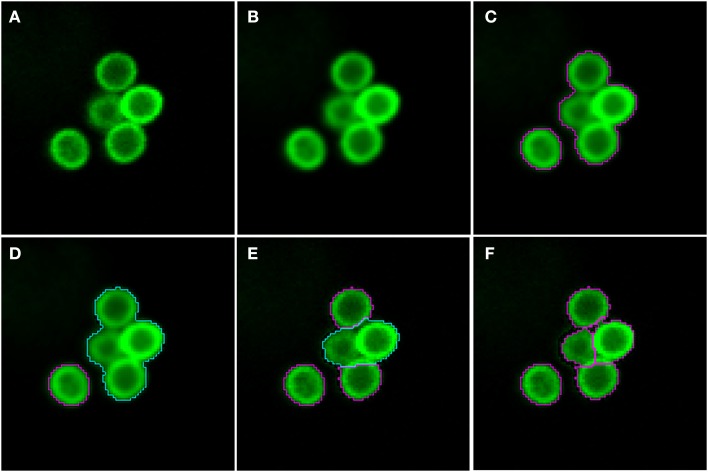
**Pre-processing and segmentation of conidia. (A)** Original scene showing a cluster of conidia and an isolated conidium. **(B)** Scene after pre-processing with a Gaussian filter. **(C)** Scene after thresholding into background and foreground indicated by magenta borders. **(D)** Distinguishing the foreground between clusters (cyan) and single conidia (magenta). Segmentation of the cluster into smaller clusters and finally single conidia after **(E)** the first and **(F)** the third iteration.

**Figure 4:**
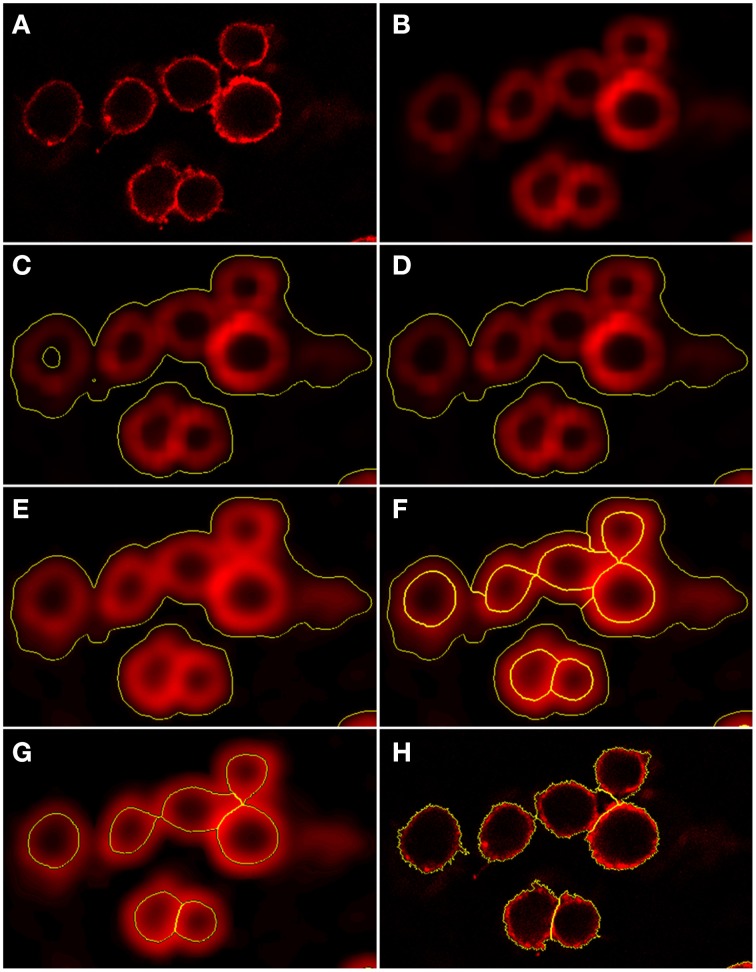
**Preprocessing and segmentation of macrophages. (A)** Original scene showing macrophages. **(B)** Scene after pre-processing with a Gaussian filter. **(C)** Scene after thresholding into background and foreground indicated by yellow borders. **(D)** Scene after merging the background enclosed by foreground into foreground. **(E)** Scene after edge detection and application of a Gaussian filter. Scene after **(F)** application of watershed segmentation and **(G)** merging of surrounding border foreground into background. **(H)** Scene after region growing enabling classification into individual macrophages.

As shown in Figure 2 (see split point 1) the green and red layer were separated and smoothed by a Gaussian filter, i.e., a low-pass filter that reduces high-frequency components in the image (Blinchikoff and Krause, 2001). The degree of smoothing by the Gaussian filter was adjusted through the parameter σ, which controls the standard deviation. This was chosen as to optimize the subsequent image segmentation: in the green layer σ = 1 px was applied and in the red layer σ = 5 px was used. For the green layer a relatively small σ was sufficient as the conidia were intensity-wise homogeneous and well separated from the background (see Figure 1A). In contrast, the higher value of σ applied on the red layer alleviated conspicuous discontinuities stemming from the staining of macrophages. Blurring helped bridging these discontinuities in macrophage boundaries and consequently improved the subsequent segmentation. Preprocessing of the blue layer, which exclusively indicated non-phagocytosed conidia, was not required, because segmentation was not applied to this layer. The calcofluor white signal, represented by the pixel intensities in this layer, was used for classification of conidia as phagocytosed or not. Figures 3A,B, 4A,B show the smoothing effects on the green and red layers, respectively; this effect was very prominent for macrophages in the red layer.

#### Segmentation

#### Segmentation of conidia

Segmenting conidia on the green layer comprised two consecutive phases. In the first phase, indicated by split point 2 in Figure 2, the image was segmented into background and foreground (conidia candidates) using clustering-based thresholding (Sezgin and Sankur, 2004). The threshold, *T*_c_, was automatically computed by a combination of intensity histogram-based measures and homogeneity measurements of segmented objects (Pal and Pal, 1993). An example of this thresholding procedure is shown in Figures 3B,C. Note that the resulting foreground objects are either single conidia, cluster of conidia or debris, which were further distinguished in the second phase of conidia segmentation. After thresholding, a morphological closing operation was performed (Gonzalez and Woods, 2006) to avoid having background enclosed in conidia as this is not a biologically realistic scenario. At the heart of identifying individual foreground objects is the *watershed segmentation*, which is a standard method for segmenting objects based on seed points that lie inside the desired objects. Starting from these seed points, it can be imagined that the intensity landscape is flooded with water filling up each object until it meets water from another object. At locations where water from different basins meets, a dam is placed that separates individual objects from each other and by that yields their segmentation (Roerdink and Meijster, 2001). In our case, seed points for each foreground object were obtained by first applying a distance transform (Fabbri et al., 2008) to the local maxima in the intensity landscape.

In the second phase, a foreground object was considered to be a cluster if its area, *A*_*cl*_ ≥ *A*^*min*^_*cl*_ = 275 px, otherwise it was considered to be a single conidium. To split a cluster into single conidia, we iteratively applied the distance transform (Fabbri et al., 2008) on the cluster to obtain seed points that were then used in the subsequent watershed (see Figure 3E). The seed points were chosen to be the local maxima obtained from the distance transform. Each time before watershed segmentation was carried out, the considered cluster was shrunk to facilitate segmentation of individual conidia. The shrinking procedure was realized by discarding pixels from the old border that have intensity below a given threshold. Before watershed segmentation was applied, all clusters were shrunk with threshold *T*^*global*^_*shrink*_ = 40 and after that, individual clusters were handled by initializing *T*^*cl*^_*shrink*_ as the minimal intensity of a specific cluster under consideration. The individual threshold was iteratively updated by *T*^*cl*^_*shrink*_ ← *T*^*cl*^_*shrink*_ + *I*_*inc*_, where *I*_*inc*_ = 10 was set during the engineering process. The iteration for splitting clusters into single conidia stopped when all segments in the original cluster had area *A*_*cl*_ < *A*^*min*^_*cl*_. The shrinking procedure was required to enhance the performance of watershed segmentation with regard to (i) identifying single conidia in clusters and (ii) correcting for a single conidium that was previously erroneously identified as a cluster. In the first case, the shrinking supported the determination of the borders of single conidia, which was necessary to identify suitable seeds for watershed segmentation. The shrinking excluded halos and reflections, which were especially strong for large clusters, and yielded seeds located on the actual conidia. Without shrinking, some seeds would have been placed with high likelihood on halos and reflections, which would lead to over-segmentation of a cluster. In the second case, the object was reduced in area below the threshold *A*^*min*^_*cl*_. This procedure corrected for single conidia with areas above *A*^*min*^_*cl*_ that can occur due to a halo caused by reflections or due to another out-of-focus conidium in its close proximity. The iteration automatically stopped when no objects were classified as clusters.

Image objects with area *A*_*c*_ ≤ *A*^*min*^_*c*_ = 100 px, or roundness ρ ≥ ρ^*max*^_*c*_ = 1.2 were discarded, because the engineering process revealed that these were most likely artifacts. Here, roundness was measured as ρ = ε^*max*^_*v*_ − ε^*min*^_*v*_, where ε^*max*^_*v*_ is the major radius of the smallest enclosing ellipse of image object *v* and ε^*min*^_*v*_ is the minor radius of the largest ellipse enclosed by *v*. Accordingly, ρ ∈ [0, ∞) and for ρ = 0 the object has a perfectly circular shape. Additionally, image objects of mean green intensity *I*_*c*_ ≤ *I*^*min*^_*c*_ = 20 were also discarded, because they were in fact out of focus. Moreover, image objects were discarded if the ratio of the long over the short main axis was bigger than 2. The iterative segmentation and exclusion of debris were the processes of split point 3 in Figure 2. An example for the segmentation of conidia in a cluster is shown in Figures 3D–F.

#### Segmentation of macrophages

The antibody-stained macrophages in the red layer were segmented in a way similar to the segmentation of conidia although three distinct phases had to be distinguished in this case: (i) thresholding, (ii) watershed segmentation and (iii) morphology-based macrophage identification. Firstly, the preprocessed layer of macrophages was segmented into background and foreground (split point 4 in Figure 2) using an automatic threshold applied to the preprocessed layer. This threshold was multiplied by a factor *f* = 0.3 in order not to lose any macrophage signal, as shown in Figures 4B,C. The resulting background with intensity below threshold consisted of regions in-between and outside macrophages, as well as areas inside the macrophages. The thresholded foreground consisted of macrophages and possibly adjacent background with pixels of high intensities due to halo effects and smoothing. Next, background segments enclosed by foreground segments were merged into the foreground. This ensured that the application of watershed segmentation performed well, because the intensity minima in these enclosed segments were used as seeds for watershed segmentation (see Figures 4C,D).

Secondly, the foreground had to be divided into regions corresponding to individual macrophages or unwanted background. To achieve this we applied an edge detection filter and then smoothed the foreground with a Gaussian filter of width σ = 9 px and applied watershed segmentation (see Figures 4E,F). Finally we identified the segments given by watershed segmentation as either macrophages or background. We could distinguish between two types of background segments according to their positions: macrophage-adjacent background segments that are bordering the background determined in the thresholding phase of segmentation and background segments that were completely enclosed by macrophages and had no contact with the background of the first phase. All macrophage-adjacent background segments were merged with the background from the thresholding, see Figure 4F. However, the enclosed background segments could not be classified in this way, because they had no contact with the background of the thresholding and determining whether these were macrophages or background required morphology-based macrophage identification using a rule-based classifier. The macrophage identification rules were based on the object's shape and brightness. The brightness β is the average intensity over the object's pixels and we consider the shape properties roundness ρ and shape index ς. The shape index describes the smoothness of an object border and is defined as ς = *b*_*v*_/(4$\sqrt{P}$_*v*_), where *b*_*v*_ is the border length of image object *v* and *P*_*v*_ is the number of all pixels forming this object. This implies ς ∈ [1, ∞) and ς = 1 indicates perfectly smooth borders. Together with the roundness ρ, these three macrophage features are considered separately as well as in various combinations to achieve optimal segmentation results. Note that we are considering two separate populations of segments when applying this ruleset, namely segments that are in contact with the image border and those which are not. For segments in contact with the border the measures have different thresholds when deciding whether they are macrophages or background.

The borders of the resulting macrophage segments are mostly located at the inner borders of macrophages in the image (see Figure 4G). To obtain the outer border we apply a region growing algorithm that first is expanding the border a fixed number of pixels in the radial direction. The engineering process revealed that two pixels was a suitable value for this growing process. Subsequently the region was grown pixel-wise in the radial direction until a step was made with an intensity drop by more than 30%. The growing process was restricted by a roundness condition preventing the macrophage segment to be extended into neighboring macrophages. This condition was expressed by the roundness ρ^*^_m_ ≤ ρ_*m*_ +0.01, where ρ_*m*_ is the initial roundness and ρ^*^_m_ is the roundness after growing. Finally, macrophage segments with area *A*_*m*_ below the threshold value *A*^*min*^_*m*_ = 2400 px were discarded because these did most likely represent artifacts (see split point 5 in Figure 2). Examples of final segmentation results are shown in Figure 4H.

#### Classification

The segmentation of image objects was followed by their classification. In particular, for each conidium we had to determine whether or not it was phagocytosed, and if not phagocytosed whether or not it was adherent to a macrophage. To distinguish between phagocytosed and non-phagocytosed conidia, we exploited the information provided from the differential staining of conidia. For each conidium, the number of calcofluor white signal was measured by computing the average blue intensity, *I*^*blue*^_*c*_, where a lower (higher) value than the threshold intensity *T*^*blue*^_*c*_ indicated that the conidium was phagocytosed (non-phagocytosed) (split point 6 in Figure 2). By validation of the automated image analysis in comparison with a manual analysis, we inferred that *T*^*blue*^_*c*_ = 37 is a suitable threshold value.

**Figure 5:**
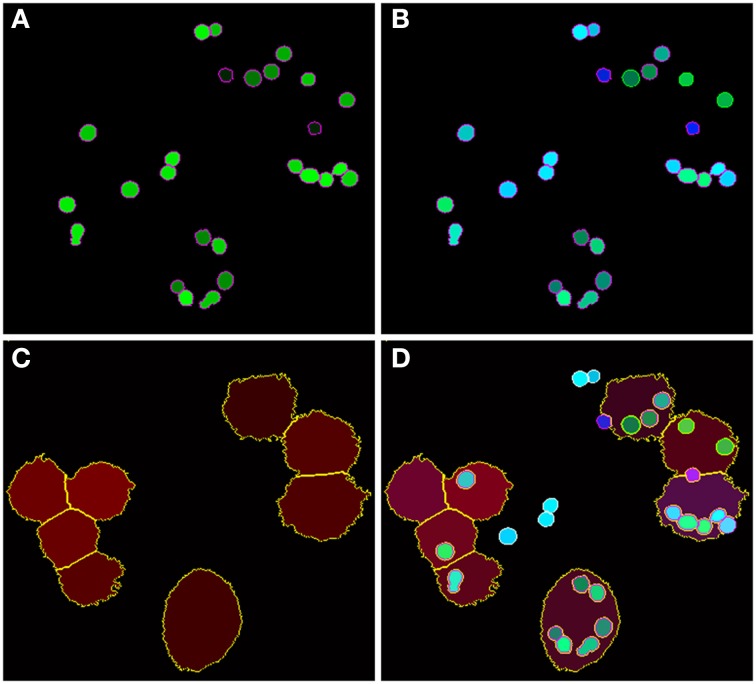
**Final image analysis result of the scene in Figure 1. (A)** All conidia and **(B)** conidia after classification as being phagocytosed (green border) or non-phagocytosed (magenta border). **(C)** Segmented macrophages. **(D)** Overlay of all image layers showing the final result of image analysis.

Next, for non-phagocytosed conidia, we differentiated between adherent and non-adherent conidia based on the relative position of conidia and macrophages (see split point 7 in Figure 2). To this end we computed the relative common border,

$$\psi =\frac{\sum_{m\in N_{c}}b(c,m)}{b_{c}},$$

with *b*(*c*, *m*) the length of the common border for objects *c* (conidium) and *m* (macrophages). *N*_*c*_ denotes the set of neighboring macrophages *m* relative to the conidium *c* and *b*_*c*_ denotes the border length of this conidium. It follows that Ψ ∈ [0, 1], where Ψ = 0 implies that no common border exists between the conidum and a macrophage. In this case the conidium was not adherent to macrophages, whereas for Ψ>0 the conidium had some common border with at least one macrophage and was therefore considered as being adherent. Moreover, we could use this equation to associate a specific conidium with a specific macrophage. A typical example of an image after performing classification is shown in Figure 5.

In close analogy to the above procedure, we could distinguish between an isolated conidium and conidia that occurred in aggregates. In this case, Ψ was computed among conidia and it was checked for common boundaries among them.

#### Statistical analysis

We performed statistical tests to evaluate the statistical significance of our results using the Wilcoxon rank-sum test (Wilcoxon, 1945) and indicated the range of the *p*-value by n.s. for non-significant (*p* ≥ 0.05), ^*^ for *p* < 0.05, ^**^ for *p* < 0.01, and ^***^ for *p* < 0.001. Distributions of data points were represented by notched box plots (Krzywinski and Altman, 2014) representing the mean value (star), the median (horizontal line), boxes containing 25% of data points above and beyond the median, whiskers excluding 2% of data points above and beyond as possible outliers. Notches represent the 95% confidence interval for their respective medians (Chambers, 1983).

## Results

In this section, we first present results on the validation of the algorithm for the automated image analysis, which was done separately for macrophage segmentation and conidia identification, i.e., involving their segmentation and classification. Next, we provide the results from the quantification of the images in terms of the distribution of conidia over macrophages. Finally, the phagocytosis of conidia as well as their aggregation were quantitatively evaluated.

### Automated image analysis algorithm reaches high performance measures

We validated our algorithm for the automated image analysis by comparison with an analysis that was carried out manually. To this end, a set of 24 images—i.e., 20% of the total number of images—were chosen randomly, such that each technical replicate was represented by three images. The visual inspection by experts was considered to be the ground truth, and the notions true positives (*TP*), false positives (*FP*) and false negatives (*FN*) were used accordingly to compute standard performance measures for binary classifications. The sensitivity is defined by

$$S=\frac{TP}{TP+FN},$$

whereas the precision is associated with the ratio

$$P=\frac{TP}{TP+FP}$$

and the accuracy is given by

$$A=\frac{TP}{TP+FP+FN},$$

where we set true negatives (*TN*) equal to zero, because these cannot occur in the current setting. All three performance measures can take values between 0 and 1, where high values indicate high performance with regard to the respective measure. The validation of macrophage segmentation as well as the identification of conidia revealed high performance measures and the results are summarized in Table 1.

**Table 1:** **Validation of conidia identification and macrophage segmentation**.

**Process**	**Number**	***TP***	***FP***	***FN***	**S [%]**	**P [%]**	**A [%]**
Macrophage segmentation	1130	1091	39	58	94.9	96.5	91.8
Conidia identification	2745	2697	48	76	97.2	98.2	95.6

Macrophage segmentation: true positives, TP, denote correctly segmented macrophages; false positives, FP, denote objects erroneously segmented as macrophages; false negatives, FN, denote macrophages missed in the segmentation. Conidia identification: true positives, TP, denote correctly segmented and classified conidia; false positives, FP, denote conidia that were falsely identified; false negatives, FN, denote conidia missed at the level of segmentation.

In the case of macrophages, *TP* are the number of correctly segmented macrophages, *FN* are those that were erroneously considered to be background, whereas regions of background that were identified as macrophages are *FP*. *FP* arise from background regions in-between macrophages that happened to have shapes with roundness and shape index similar to actual macrophages. On the other hand, *FN* arise from macrophages that were not detected by the algorithm because of highly uneven staining and/or low integrity of the macrophage boundary, or because the roundness and shape index were similar to background segments, which can occur when a macrophage is partially covered by another macrophage.

The identification of conidia refers to the combined process of conidia segmentation and classification. In particular, we focused on the classification of phagocytosed vs. non-phagocytosed conidia. Thus, *TP* are the number of conidia which were correctly segmented and classified as either phagocytosed or non-phagocytosed. The conidia that were falsely identified—i.e., either erroneously segmented as conidia or falsely classified—were counted as *FP*, whereas *FN* are conidia that were missed at the level of segmentation.

In passing we note that we checked the technical and biological replicates of images for the MOI. In the experimental protocol, where the MOI was initially set to 7, several washing steps were required that affect the number of conidia relative to the number of macrophages. This loss of non-phagocytosed and mostly non-adherent conidia could not be avoided, however, we checked that the ultimate MOI was comparable in the images and found 2.42 ± 0.64 for the wild-type ATCC strain and 2.26 ± 0.76 for the CEA10 strain.

### Distribution of conidia over macrophages yields full quantification of image data

**Figure 6:**
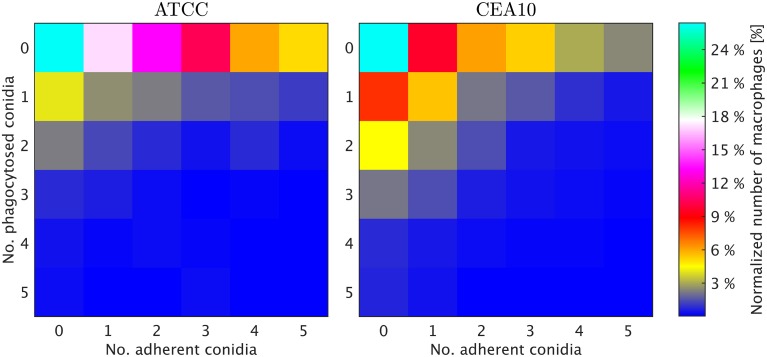
**Two-dimensional probability distributions of adherent and phagocytosed conidia over macrophages for the two** ***A. fumiatus*** **strains ATCC and CEA10**.

**Figure 7:**
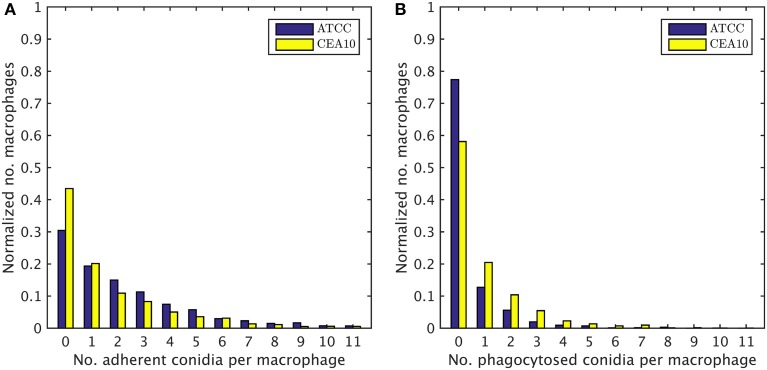
**One-dimensional probability distributions for a macrophage to have a certain number of (A) adherent and (B) phagocytosed conidia**.

We exploited the segmentation of macrophages to study the distribution of phagocytosed and adherent conidia over macrophages and the correlation between them. In Figure 6 the probability distribution of macrophages as function of the number of adherent and the number of phagocytosed conidia is shown. This distribution contains the full information about the phagocytosis seen in the images. Already at this stage, qualitative differences between the strains when confronted with macrophages were detected. For example, comparing adherence of conidia to macrophages, it was observed this occurred less frequently for the strain CEA10 than for ATCC. Approximately 25% of the macrophages had three or more adherent CEA10 conidia while more than 35% had three or more adherent ATCC conidia. Conversely, about 80% of the macrophages confronted with the ATCC strain did not phagocytose, whereas approximately 60% of the macrophages did not phagocytose when confronted with the CEA10 strain. This tendency yielded the qualitative information that immune cells responded more vigorously to CEA10 than to the ATCC strain. By summing the two-dimensional distribution along its individual axes we obtained the distribution of phagocytosed conidia and the distribution of adherent conidia (see Figure 7). These distributions confirmed the impression from Figure 6 as the distribution of adherent conidia for ATCC was clearly shifted to higher conidia numbers compared to CAE10 (see Figure 7A), while the opposite was found in the case of phagocytosis (see Figure 7B).

### Quantification of phagocytosis events can be studied from different viewpoints

In previous studies, different scalar measures were used to quantify the phagocytosis process, which was partly a consequence of the fact that not all cells and interactions could be resolved (Sano et al., 2003; Mech et al., 2011; Kraibooj et al., 2014). We here present a comprehensive collection of various measures and compute them for comparison, i.e., from either the viewpoint of conidia or macrophages or from a combination of both viewpoints.

The conidia point of view is represented by the phagocytosis ratio that compares all phagocytosed conidia to all macrophage-associated conidia,

$$\varphi_{c}=\frac{N_{c}^{phag}}{N_{c}^{phag}+N_{c}^{adh}}.$$

**Figure 8:**
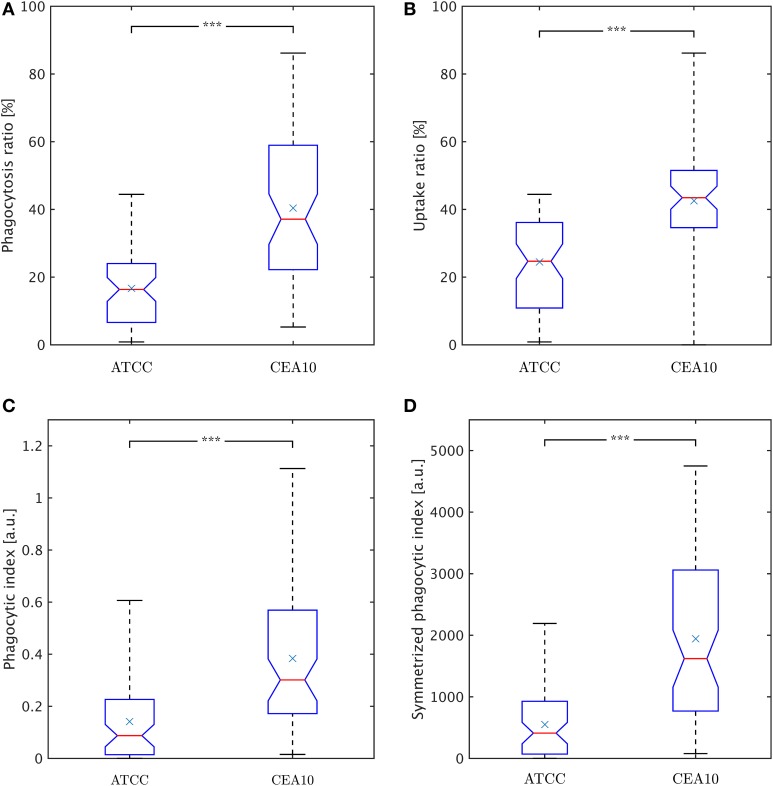
**Comparison of phagocytosis measures between the two** ***A. fumiatus*** **strains ATCC and CEA10. (A)** Phagocytosis ratio φ_*c*_. **(B)** Uptake ratio φ_*m*_. **(C)** Phagocytic index φ_*i*_. **(D)** Symmetrized phagocytic index φ^*sym*^_*i*_. See the text for details.

Here, *N*^*phag*^_*c*_ denotes the number of phagocytosed conidia and *N*^*adh*^_*c*_ is the number of adherent conidia. It should be noted that this viewpoint intentionally neglects conidia that were not phagocytosed and not adherent to macrophages, because those conidia may have never been in contact with macrophages during the experiment. The phagocytosis ratio for the two strains is compared in Figure 8A, where the difference in phagocytosis, which was qualitatively discussed based on the distributions in Figures 6, 7, was tested for statistical significance.

The macrophage point of view is expressed by the uptake ratio (Sano et al., 2003), which is the ratio of phagocytosing macrophages to all macrophages,

$$\varphi_{m}=\frac{N_{m}^{phag}}{N_{m}},$$

where *N*^*phag*^_*m*_ denotes the number of phagocytosing macrophages and *N*_*m*_ the total number of macrophages. Figure 8B shows the uptake ratio for both strains. Although the phagocytosis ratio and the uptake ratio give a good idea about phagocytosis events in experiments, the picture is more complete when studying the mutual effect of both points of view. For this, the phagocytic index,

$$\varphi_{i}=\frac{N_{c}^{phag}}{N_{m}}\;\cdot \;\varphi_{m},$$

corresponds to the product of the number of phagocytosed conidia per macrophage and the uptake ratio (Sano et al., 2003). Figure 8C shows the phagocytic index of both strains. Similarly, it could be argued for combining the uptake ratio with the phagocytosis ratio,

$$\varphi_{i}^{sym}=\varphi_{c}\;\cdot \;\varphi_{m},$$

where the number of macrophage-associated conidia and the number of macrophages entercontribute in a more symmetric fashion and which is therefore referred to as symmetrized phagocytic index. This measure is presented in Figure 8D for comparison with the other measures. All measures and distributions point toward a higher degree of phagocytosis for the CEA10 strain compared to ATCC.

### Aggregation observed for adherent but not for non-adherent conidia

**Figure 9:**
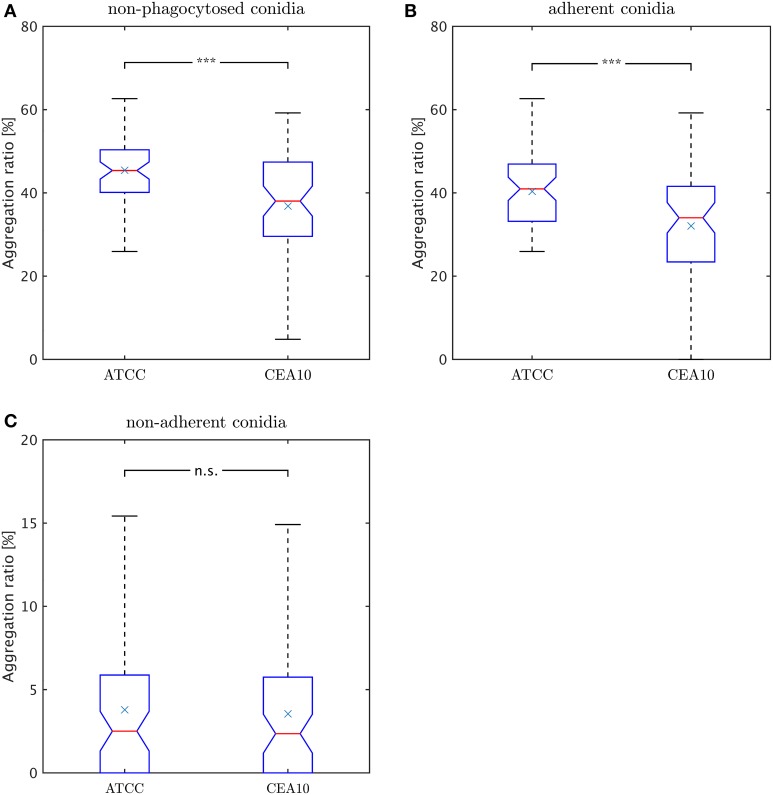
**Comparison of aggregation ratio, γ**_***r***_**, of non-phagocytosed conidia between the two** ***A. fumiatus*** **strains ATCC and CEA10**. Aggregation ratio relative to **(A)** all non-phagocytosed conidia, **(B)** adherent conidia, and **(C)** non-adherent conidia.

Rather than occurring as isolated cells, conidia were often observed to cluster in aggregates. We consider image objects to be aggregated if they have common borders. As explained in subsection Classification of the Materials and Methods section in the context of macrophage-adherence by a conidium, we computed common borders between conidia to identify such clusters. The aggregation ratio is defined by,

$$\gamma_{r}=\frac{N_{c}^{agg}}{N_{c}^{non-phag}},$$

where *N*^*agg*^_*c*_ denotes all non-phagocytosed conidia which are aggregated and *N*^*non*−*phag*^_*c*_ is the number of all conidia which are non-phagocytosed. Thus, we did not account for phagocytosed conidia, because their visual aggregation might solely be appearing due to spatial constraints in the macrophage. Since non-phagocytosed conidia could be adherent or non-adherent, we distinguished between the aggregation ratio for non-adherent conidia and for adherent conidia. For this purpose, we distinguished between adherent clusters and non-adherent clusters and we applied the same formula. In Figure 9A we observe a higher aggregation ratio for ATCC compared to CAE10 when considering all non-phagocytosed conidia. However, when we divided this population into adherent and non-adherent conidia (see Figures 9B,C) it was revealed that the difference in aggregation between the two strains was only present in the adherent conidia. As we have already demonstrated that ATCC conidia were adherent to macrophages to a higher degree than CEA10, whereas the latter is more likely to be phagocytosed, it may be argued that the difference in aggregation was merely the consequence of spatial limitation on the surface of macrophages. For the population of conidia that are neither phagocytosed nor adherent to a macrophage, both strains showed a very low aggregation ratios with no significant difference between them.

## Discussion

Confrontation assays are commonly used today to quantify host-pathogen interactions between immune cells and pathogenic cells. In contrast to techniques based on flow cytometry, approaches based on microscopy images do provide a richer amount of information, e.g., on spatial correlations and morphological properties of cells. However, the information gained by image-based approaches is typically foiled by a tedious and error-prone manual analysis of the data. In this work, we addressed this drawback and developed a computer algorithm that performs the image analysis automatically and that opens up the possibility for high-throughput screening while retaining the full information content of the images. In fact, for the microscopy images analyzed in the present study, we achieved computation times per image of about 1 min, which was more than one order of magnitude lower than was required for a manual analysis.

We performed a rigorous validation of the algorithm and obtained high performance measures for the overall sensitivity (96.6%), precision (97.8%), and accuracy (94.5%), which are reasonable values in the light of the high variation of image objects. We identified the main source of errors to be the similarity in shape and intensity properties between image objects and background regions. For example, this concerned background regions that were completely surrounded by macrophages or conidia, or macrophages that were of too low signal as a result of insufficient staining, or clusters with slightly superimposed conidia that could not be correctly identified because of their effectively smaller cluster size.

Algorithms for the automated image analysis of confrontation assays, especially in the context of fungal pathogens interacting with immune cells, have been developed before (Mech et al., 2011, 2014; Kraibooj et al., 2014; Schäfer et al., 2014). The most important progress of the novel algorithm presented here concerns the successful segmentation of macrophages. This was not achieved previously, because it was complicated by the occasionally interrupted staining of the macrophage surface, but is a necessary prerequisite for the comprehensive quantification of these phagocytosis assays. Thus, having achieved this crucial progress in the present work, the task of quantifying this type of phagocytosis assays was now comprehensively solved, because spatial and functional information on the interaction between all cells in the assay is now accessible. For example, we demonstrated that macrophages involved in the phagocytosis process can be detected and whether macrophage-associated conidia were really phagocytosed or just adherent. This enables building up a two-dimensional distribution of conidia over the macrophages from which other measures describing the immune response can be derived.

Interestingly, different measures have been proposed for the quantification of biological processes such as phagocytosis events. Taking either the viewpoint of the pathogens or the immune cells, we here computed the phagocytosis ratio φ_*c*_ (Mech et al., 2011; Kraibooj et al., 2014) and the uptake ratio φ_*m*_ (Sano et al., 2003), respectively. A quantity that combines both viewpoints is commonly referred to as phagocytic index φ_*i*_ (Sano et al., 2003), for which a symmetrized measure φ^*sym*^_*i*_ = φ_*c*_ · φ_*m*_ was proposed here. Obviously, all these measures will provide different absolute numbers and can be used in the comparison of different strains. More importantly, they have to be interpreted with some care, which is in particular true for the measures with the combined viewpoint. This is a consequence of the fact that these measures involve a multiplication of two factors. For example, as can be easily demonstrated for the symmetrized phagocytic index, a high value of the phagocytosis ratio φ_*c*_ and a low value of the uptake ratio φ_*m*_ yield a value for φ^*sym*^_*i*_ that can be identical for low φ_*c*_ and high φ_*m*_ and by that masks important differences in the underlying biology. Therefore, we conclude that, rather than representing the immune response by a single scalar measure, interpretation of the confrontation assay should be inferred from a comparison of the distribution of pathogens over immune cells. If the confrontation assays contain more than two different cell types, the distribution can be extended to higher dimensions as long as sufficient data are available.

In the present study, conidia of strain CEA10 showed a significantly higher phagocytosis ratio than ATCC. This was not only confirmed for the viewpoint of conidia by the phagocytosis ratio and of macrophages by the uptake ratio, but also by the combined viewpoint of conidia and macrophages in terms of the phagocytic index and the symmetrized phagocytic index. From the quantities of these measures, it can be concluded that the experiments were neither limited by the saturation of macrophages with phagocytosed conidia nor by the depletion of non-phagocytosed conidia. Thus, the significant differences in all measures indicated that the process of conidia recognition and uptake was generally more effective for CEA10 than for ATCC. In particular, this could be concluded from the fact that not only the percentage of phagocytosed conidia was higher for CEA10 than for ATCC, but also the percentage of phagocytosing macrophages. The conclusion that the initiation of phagocytosis was less effective for ATCC was also in line with the observation that the number of ATCC conidia that were adherent to macrophages were higher compared to CEA10 conidia. Furthermore, we could exclude that differences in the phagocytosis of conidia were due to the aggregation of conidia, because no significant differences in the aggregation ratio were observed for the non-adherent conidia of the two strains.

Previous studies on an embryonated egg model and in mice showed that CEA10 was more virulent than ATCC (Jacobsen et al., 2010; Heinekamp and Brakhage, 2012). The question remains if the difference in virulence is directly correlated to the observed difference in phagocytosis ratio. In a similar study on *Lichtheimia corymbifera* it was also shown that a more virulent strain was more effectively phagocytosed (Kraibooj et al., 2014). In case the spores are able to inhibit killing after being phagocytosed they could use macrophages as a survival niche and escape from the phagocyte by germination (Amin et al., 2014). However, further experiments would have to be performed to prove this hypothesis.

We expect that our novel algorithm for fully automated image analysis will be of importance in future research for several reasons. Firstly, it paves the way for comparative high-throughput screening of mutant collections and their comprehensive quantification. Secondly, the algorithm is generally applicable to assays of cells with close-to circular morphology and can be straightforwardly extended to assays for more than two different cell types. Thirdly, the results achieved here form a quantitative data base for the development of mathematical models that enable realistic simulations of biological processes on the computer. Image-based systems biology is a modern field of research (Medyukhina et al., 2015) and has a plethora of applications, for example, providing image-derived techniques for differentiating between cell colocalization and random positioning of cells (Mokhtari et al., 2015) or simulating virtual infection models for *A. fumigatus* infection (Tokarski et al., 2012; Pollmächer and Figge, 2014).

## Author contributions

Conception and design of the investigation and work: CS, AB, MTF. Contribution of materials and computational resources: AB, MTF. Imaging experiments: HS. Data processing, development and application of the computer algorithm: KK, CS. Evaluation and analysis of the results: KK, HS, CS, AB, MTF. Drafting the manuscript and revising it critically for important intellectual content and final approval of the version to be published: KK, HS, CS, AB, MTF. Agreement to be accountable for all aspects of the work in ensuring that questions related to the accuracy or integrity of any part of the work are appropriately investigated and resolved: KK, HS, CS, AB, MTF.

### Conflict of interest statement

The authors declare that the research was conducted in the absence of any commercial or financial relationships that could be construed as a potential conflict of interest.
