# Supplementary material for: Automated quantification of the phagocytosis of Aspergillus fumigatus conidia by a novel image analysis algorithm
Source: Front Microbiol. 2015 Jun 9;6:549. doi: 10.3389/fmicb.2015.00549 (PMC4460560; doi:10.3389/fmicb.2015.00549)
Supplement: Supplementary file 1 [file DataSheet1.PDF]

Supplementary Material:

Automated quantification of the phagocytosis of *Aspergillus fumigatus* conidia by a novel image analysis algorithm

Kaswara Kraibooj[1,3,†], Hanno Schoeler[2,3,†], Carl-Magnus Svensson[1], Axel A. Brakhage[2,3] and Marc Thilo Figge[1,3,*]

[1]Applied Systems Biology, Leibniz Institute for Natural Product Research and Infection Biology – Hans Knöll Institute (HKI), Jena, Germany
[2]Department of Molecular and Applied Microbiology, Leibniz Institute for Natural Product Research and Infection Biology – Hans Knöll Institute (HKI), Jena, Germany
[3]Friedrich Schiller University Jena, Jena, Germany

[†] **These authors contributed equally to this work.**

*** Correspondence:** Marc Thilo Figge, Applied Systems Biology, Leibniz Institute for Natural Product Research and Infection Biology – Hans Knöll Institute, Beutenbergstrasse 11a, 07745 Jena, Germany. e-mail: thilo.figge@hki-jena.de

Keywords: *Aspergillus fumigatus*, alveolar macrophages, host-pathogen interaction, phagocytosis assay, automated image analysis.

1 Supplementary Data

Supplementary Data 1. Rule-set of the algorithm in the meta-language of the software *Definiens Developer XD*.

Classes:
 _Temp1
 _Temp2
 _Temp4
 background
 cluster_temp
 cluster
 conidia_adh
 conidia_nonadh
 conidia_nonphag
 conidia_phag
 link_class_adh
 link_mphag-adhC_NB
 link_mphage-phagC_NB
 link_mphage_allC_NB
 link_mphage_phagC
 mphages
 nonadhC_notknown

Process: Main:
ruleset
initialization
Reset
delete image object level: at Mphage_Level: delete
delete image object level: at Spores_Level: delete
delete links: mphages, conidia_phag at Mphage_Level: delete background, mphages, conidia_phag(all) links to any with class=any
delete links: link_class_adh, link_mphage_phagC at Mphage_Level: delete background, mphages, conidia_phag(all) links to any with class=any
delete layer: delete image layer '1_gaus'
delete layer: delete image layer '1_gaus_minmax'
delete layer: delete image layer 'L2_gaus'
delete layer: delete image layer 'distance map'
delete map: on spores_map : delete map
delete map: on mphage_map : delete map
Initialize Variables
update variable: adhC_NUM_1 *= 0
update variable: curr_round *= 0
update variable: thresh_L2 *= 0
update variable: thresh_1gaus *= 0
update variable: adhC_RATIO_2 *= 0
update variable: index2 *= 0
update variable: adhC_RATIO_3 *= 0
update variable: mphage_RATIO_2 *= 0
update variable: mphage_RATIO_3 *= 0
update variable: mphage_RATIO_4 *= 0
update variable: mphage_RATIO_1 *= 0
update variable: mphage_NUM_4 *= 0
update variable: mphage_NUM_3 *= 0
update variable: associatedC_NB_NUM *= 0
update variable: nonphagC_NUM_1 *= 0
update variable: phagC_NUM_1 *= 0
update variable: mphage_NUM_2 *= 0
update variable: mphage_Num_6 *= 0
update variable: mphage_NUM_1 *= 0
update variable: agg_NUM *= 0
update variable: agg_adh_RATIO *= 0
update variable: agg_nonadh_RATIO *= 0
update variable: agg_adh_NUM *= 0
update variable: agg_nonadh_NUM *= 0
update variable: adhC_RATIO_1 *= 0
update variable: adh_INDEX *= 0
update variable: agg_RATIO *= 0
update variable: phagC_RATIO_2 *= 0
update variable: thresh_1gaus *= 0
update variable: initial_shrink_thresh *= 0
update variable: phag_INDEX *= 0

update variable: phagC_RATIO_3 *= 0
update variable: phagC_RATIO_1 *= 0
update variable: totalC_classi_NUM *= 0
update variable: totalC_seg_NUM *= 0
update variable: phagC_NUM_2 *= 0
update variable: adhC_NUM_2 *= 0
update variable: notknownC_NUM *= 0
update variable: adhConPerMphage_RATIO *= 0
update variable: nonadhC_NUM_1 *= 0
update variable: counter *= 0
update variable: phagC_per_M_DIST *= 0
update variable: uptake_percentage_phag *= 0
update variable: uptake_percentage_adh *= 0
update variable: MOI *= 0
update variable: step_size *= 0
update variable: clust_intens_MIN *= 0
update variable: mphage_NUM_5 *= 0
update variable: curr_init_intens *= 0
update array: update array 'cluster_array': clear
update array: update array 'cluster_non-adh': clear
update array: update array 'cluster_adh': clear
Parameters
Conidia
update variable: conidium_area_MIN = 100
update variable: conidium_intensity_MIN = 20
update variable: clust_size_MIN = 275
update variable: conidium_roundness_MAX = 1.2
update variable: conidium_LW_MAX = 2
update variable: C_blue_THR = 40
update variable: step_size = 10
update variable: watershed_C_area_THR = 10
update variable: initial_shrink_thresh = 40
Mphages
update variable: mphage_border_intensity = 80
update variable: watershed_M_area_THR = 50
update variable: mphage_area_MIN = 2400
update variable: mphage_area_MAX = 40000
pre-prosessing and segmentation of conidia (*)
create Conidia Map
delete map: on spores_map : delete map
copy map: on main : copy map to 'spores_map' with scale 100%
display map: on spores_map : display map 'spores_map'
pre-processing
convolution filter: on spores_map : convolution filter (Gauss Blur, 3 x 3 x 1): 'Layer 2' => 'L2_gaus'
thresholding
automatic threshold-based segmentation
automatic threshold: on spores_map : thresh_L2=auto threshold on L2_gaus

```
          update variable: thresh_L2 *= 1.5
          multi-threshold segmentation: on spores_map :  creating 'Spores_Level': unclassified <= thresh_L2 < conidia_phag on L2_gaus
        special cases_filling holes
          find enclosed by class: unclassified at  Spores_Level: enclosed by conidia_phag: conidia_phag +
          assign class: unclassified with Area < conidium_area_MIN and Distance to scene border = 0 Pxl at  Spores_Level: conidia_phag
          merge region: conidia_phag at  Spores_Level: merge region
          grow region: _Temp2 at  Spores_Level: <- conidia_phag
          assign class: _Temp2 at  Spores_Level: conidia_phag
      Iterative segmentation_cell seperation
        initial shrinking
          pixel-based object resizing: loop: on spores_map conidia_phag with Area >= clust_size_MIN at  Spores_Level: shrink using unclassified where Layer 2<=initial_shrink_thresh
        Init
          assign class: on spores_map conidia_phag with Area >= clust_size_MIN at Spores_Level: _Temp1
        Cell Seperation
          _Temp1 at  Spores_Level
            assign class: if _Temp1 : cluster_temp
            update variable: cluster_temp at  Spores_Level: clust_intens_MIN = Min. pixel value Layer 2
            update variable: curr_init_intens = [clust_intens_MIN]
            merge region: cluster_temp at  Spores_Level: merge region
            loop: cluster_temp at  Spores_Level
              loop: do
                update variable: curr_init_intens += step_size
                pixel-based object resizing: loop: cluster_temp at  Spores_Level: shrink using unclassified where Layer 2<=curr_init_intens
                distance map: cluster_temp at  Spores_Level: distance to object border(distance map)
                watershed segmentation: cluster_temp at  Spores_Level: watershed segmentation on -distance map with fusion
                convert image objects: cluster_temp, conidia_phag at  Spores_Level: convert image objects -> Connected 2D
                assign class: on spores_map cluster_temp with Area < clust_size_MIN at  Spores_Level: conidia_phag
      Clean up
        assign class: conidia_phag with Area < conidium_area_MIN at  Spores_Level: unclassified
        assign class: on spores_map conidia_phag with Mean Layer 2 <= conidium_intensity_MIN at  Spores_Level: unclassified
        assign class: on spores_map conidia_phag with Roundness >= conidium_roundness_MAX at  Spores_Level: unclassified
        assign class: on spores_map conidia_phag with Length\Width >= conidium_LW_MAX at  Spores_Level: unclassified
```

merge region: unclassified at Spores_Level: merge region
Quantities
compute statistical value: conidia_phag at Spores_Level: totalC_seg_NUM = number
nonphag conidia and Phag conidia
assign class: on spores_map conidia_phag with Mean Layer 4 >= 37 at Spores_Level: conidia_nonphag
compute statistical value: conidia_nonphag at Spores_Level: nonphagC_NUM_1 = number
compute statistical value: conidia_phag at Spores_Level: phagC_NUM_1 = number
aggregation_non-phag
Specify Clusters
on spores_map cluster, conidia_nonphag with Number of conidia_nonphag (0) >= 1 at Spores_Level
assign class: on spores_map cluster, conidia_nonphag with Rel. border to conidia_nonphag > 0 at Spores_Level: cluster
Number of nonphagocytosed aggregated conidia
compute statistical value: cluster at Spores_Level: agg_NUM = number
Count Clusters and assign the numbers to array
on spores_map cluster at Spores_Level (*)
assign class: if cluster : cluster_temp
assign class: 10x: on spores_map cluster_temp, cluster with Rel. border to cluster_temp > 0 at Spores_Level: cluster_temp
compute statistical value: on spores_map cluster_temp at Spores_Level: counter = number
update array: on spores_map if counter > 1 : update array 'cluster_array': add: [counter]
assign class: on spores_map cluster_temp at Spores_Level: conidia_nonphag
update variable: counter = 0
pre-processing and segmentation of macrophages (*)
Create Mphage Map
delete map: on mphage_map : delete map
copy map: on main : copy map to 'mphage_map'
display map: on mphage_map : display map 'mphage_map'
Preprocessing
convolution filter: convolution filter (Gauss Blur, 25 x 25 x 1): 'Layer 1' => '1_gaus'
Segmentation
thresholding
automatic-thrshold-based segmentaiton
automatic threshold: on mphage_map : thresh_1gaus=auto threshold on 1_gaus
update variable: on mphage_map : thresh_1gaus *= 0.3
multi-threshold segmentation: on mphage_map : creating 'Mphage_Level': unclassified <= thresh_1gaus < mphages on 1_gaus
merge in-between background to foreground
find enclosed by class: on mphage_map unclassified at Mphage_Level: enclosed by mphages: _Temp1 +

remove objects: _Temp1 at Mphage_Level: remove objects into mphages (merge by shape)

assign class: unclassified with Distance to scene border = 0 Pxl and Area <= 7000 at Mphage_Level: mphages

merge region: on mphage_map mphages at Mphage_Level: merge region

watershed segmentation

watershed segmentation

pixel min/max filter (prototype): on mphage_map mphages at Mphage_Level: pixel min\max filter (prototype) (3 x 1; Diff. center to darkest): 'Layer 1' => '1_gaus_minmax'

convolution filter: on mphage_map mphages at Mphage_Level: convolution filter (Gauss Blur, 49 x 49 x 1): '1_gaus_minmax' => '1_gaus'

watershed segmentation: on mphage_map mphages at Mphage_Level: watershed segmentation on 1_gaus with fusion

convert image objects: mphages at Mphage_Level: convert image objects -> Connected 2D

merge thresholding-background with watershed background

assign class: on mphage_map mphages with Border to unclassified > 0 Pxl at Mphage_Level: _Temp1

assign class: on mphage_map _Temp1 at Mphage_Level: unclassified

merge region: on mphage_map unclassified at Mphage_Level: merge region

classifiers

border classifier

assign class: on mphage_map mphages with Distance to scene border = 0 Pxl at Mphage_Level: _Temp4

assign class: on mphage_map _Temp4 with Mean Layer 4 <= 5 and Mean Layer 1 <= 20 at Mphage_Level: unclassified

assign class: on mphage_map _Temp4 with Area >= mphage_area_MAX at Mphage_Level: unclassified

assign class: on mphage_map _Temp4 with Mean Layer 4 <= 1 at Mphage_Level: unclassified

assign class: on mphage_map _Temp4 with Roundness >= 0.8 and Shape index >= 1.8 at Mphage_Level: unclassified

assign class: on mphage_map _Temp4 with Roundness >= 1 at Mphage_Level: unclassified

assign class: on mphage_map _Temp4 with Mean Layer 1 <= 65 at Mphage_Level: _Temp1

assign class: on mphage_map _Temp1 with Roundness >= 0.65 and Shape index >= 1.2 at Mphage_Level: unclassified

assign class: on mphage_map _Temp1 with Roundness >= 0.6 and Shape index >= 1.41 at Mphage_Level: unclassified

assign class: on mphage_map _Temp1, _Temp4 at Mphage_Level: mphages

inside classifier

assign class: on mphage_map mphages with Distance to scene border > 0 Pxl at Mphage_Level: _Temp4

assign class: on mphage_map _Temp4 with Area >= mphage_area_MAX at

Mphage_Level: unclassified
assign class: on mphage_map _Temp4 with Mean Layer 4 <= 1 at Mphage_Level: unclassified
assign class: on mphage_map _Temp4 with Mean Layer 4 <= 5 and Mean Layer 1 <= 20 at Mphage_Level: unclassified
assign class: on mphage_map _Temp4 with Mean Layer 4 <= 2 and Mean Layer 1 <= 42 at Mphage_Level: unclassified
assign class: on mphage_map _Temp4 with Mean Layer 4 <= 5 and Shape index >= 1.4 at Mphage_Level: unclassified
assign class: on mphage_map _Temp4 with Mean Layer 4 <= 4 and Shape index >= 1.28 at Mphage_Level: unclassified
assign class: on mphage_map _Temp4 with Mean Layer 4 <= 3 and Roundness >= 0.55 at Mphage_Level: unclassified
assign class: on mphage_map _Temp4 with Roundness >= 0.51 and Shape index >= 1.4 at Mphage_Level: unclassified
assign class: on mphage_map _Temp4 with Roundness >= 0.6 and Shape index >= 1.3 at Mphage_Level: unclassified
assign class: on mphage_map _Temp4 with Roundness >= 0.73 and Shape index >= 1.57 at Mphage_Level: unclassified
assign class: on mphage_map _Temp4 with Roundness >= 0.72 and Shape index >= 1.47 at Mphage_Level: unclassified
assign class: on mphage_map _Temp4 at Mphage_Level: mphages
Smoothing The Borders of Macrophages
pixel-based object resizing: 2x: mphages at Mphage_Level: grow into all where Layer 1>=10
chessboard segmentation: on mphage_map unclassified at Mphage_Level: chess board: 1
on mphage_map mphages at Mphage_Level
update variable: if mphages : curr_round = [Roundness]+0.01
grow region: on mphage_map mphages with Roundness <= curr_round at Mphage_Level: <- unclassified Mean Layer 1 ratio PPO (0) >= 0.7 (*)
cleanup
merge region: on mphage_map unclassified at Mphage_Level: merge region
assign class: on mphage_map mphages with Area <= mphage_area_MIN and Distance to scene border > 0 Pxl at Mphage_Level: unclassified
assign class: on mphage_map unclassified at Mphage_Level: background
find enclosed by class: on mphage_map background, unclassified at Mphage_Level: enclosed by mphages: _Temp1 +
remove objects: on mphage_map _Temp1 at Mphage_Level: remove objects into mphages (merge by shape)
merge region: on mphage_map background at Mphage_Level: merge region
linking
create links: on mphage_map mphages with Distance to scene border > 0 Pxl at Mphage_Level: *link link_mphage_allC_NB to any if class=conidia_nonphag, conidia_phag, shift=0x0x0x0, overlap>=0%
create links: on mphage_map mphages at Mphage_Level: *link link_mphage_phagC to any if class=conidia_phag, shift=0x0x0x0, overlap>=0%
create links: on mphage_map mphages with Distance to scene border > 0 Pxl at

Mphage_Level: *link link_mphage-phagC_NB to any if class=conidia_phag, shift=0x0x0x0, overlap>=0%
create links: on mphage_map mphages at Mphage_Level: *link link_class_adh to any if class=conidia_nonphag, shift=0x0x0x0, overlap>=0%
create links: on mphage_map mphages with Distance to scene border > 0 Pxl at Mphage_Level: *link link_mphag-adhC_NB to any if class=conidia_nonphag, shift=0x0x0x0, overlap>=0%
update variable: on mphage_map mphages at Mphage_Level: adh_per_mphage_noborder = Number of linked objects Number of Linked via link_class_adh(all) within dist=999999, excl. current object
update variable: on mphage_map mphages at Mphage_Level: phag_per_mphage_pos_noborder = Number of linked objects Number of Linked via link_mphage_phagC(all) within dist=999999, excl. current object
adh, non-adh&non-known
assign class: on spores_map conidia_nonphag with Number of linked objects Number of Linked via link_class_adh(all) within dist=999999, excl. current object = 0 at Spores_Level: conidia_nonadh
assign class: on spores_map conidia_nonphag at Spores_Level: conidia_adh
assign class: on spores_map conidia_nonadh with Distance to scene border = 0 Pxl at Spores_Level: nonadhC_notknown
aggregation
Aggregation ratio of all non-phag spores
[on mphage_map conidia_nonphag at Mphage_Level]
update variable: conidia_nonphag neighbour image object: agg_NUM += 1
update variable: agg_RATIO = ([agg_NUM]\[nonphagC_NUM_1])*100
update variable: agg_RATIO = ([agg_NUM]\[nonphagC_NUM_1])*100
clusters_adh
init
update variable: counter = 0
assign class: on spores_map cluster at Spores_Level: cluster_temp
Specify Clusters
on spores_map cluster, conidia_adh with Number of conidia_adh (0) >= 1 at Spores_Level
assign class: on spores_map cluster, conidia_adh with Rel. border to conidia_adh > 0 at Spores_Level: cluster
Number of nonphagocytosed aggregated conidia
compute statistical value: cluster at Spores_Level: agg_adh_NUM = number
Count Clusters and assign the numbers to array
on spores_map cluster at Spores_Level
assign class: if cluster : cluster_temp
assign class: 10x: on spores_map cluster_temp, cluster with Rel. border to cluster_temp > 0 at Spores_Level: cluster_temp
compute statistical value: on spores_map cluster_temp at Spores_Level: counter = number
update array: if counter > 1 : update array 'cluster_adh': add: [counter]
assign class: on spores_map cluster_temp at Spores_Level: conidia_adh
update variable: counter = 0
clusters_nonadh

init
update variable: counter = 0
assign class: cluster at Spores_Level: cluster_temp
Specify Clusters
on spores_map cluster, conidia_nonadh with Number of conidia_nonadh (0) >= 1 at Spores_Level
assign class: on spores_map cluster, conidia_nonadh with Rel. border to conidia_nonadh > 0 at Spores_Level: cluster
Number of nonphagocytosed aggregated conidia
compute statistical value: cluster at Spores_Level: agg_nonadh_NUM = number
Count Clusters and assign the numbers to array
on spores_map cluster at Spores_Level
assign class: if cluster : cluster_temp
assign class: 10x: on spores_map cluster_temp, cluster with Rel. border to cluster_temp > 0 at Spores_Level: cluster_temp
compute statistical value: on spores_map cluster_temp at Spores_Level: counter = number
update array: on spores_map if counter > 1 : update array 'cluster_non-adh': add: [counter]
assign class: on spores_map cluster_temp at Spores_Level: conidia_nonadh
update variable: on spores_map : counter = 0
Quantities
with borders' Mphages
Numbers
total number of conidia
update variable: totalC_classi_NUM = [phagC_NUM_1]+[nonphagC_NUM_1]
total number of mphages
compute statistical value: on mphage_map mphages at Mphage_Level: mphage_NUM_1 = number
number of phagozytosed spores including borders' Mphage
compute statistical value: on spores_map conidia_phag at Spores_Level: phagC_NUM_1 = number
number of adherent conidia including borders
compute statistical value: on spores_map conidia_adh at Spores_Level: adhC_NUM_1 = number
number of non-adherent conidia excluding borders' ones
compute statistical value: on spores_map conidia_nonadh with Distance to scene border > 0 Pxl at Spores_Level: nonadhC_NUM_1 = number
Ratios
Phagocytosis ratio by color including borders
update variable: on spores_map : phagC_RATIO_1 = ([phagC_NUM_1]\([phagC_NUM_1]+[adhC_NUM_1]))*100
Phagocytosis ratio by color including borders (to all spores)
update variable: on spores_map : phagC_RATIO_3 = [phagC_NUM_1]\([phagC_NUM_1]+[nonphagC_NUM_1])*100

Adhesion ratio including borders
update variable: adhC_RATIO_1 = ([adhC_NUM_1]\([nonphagC_NUM_1]))*100
Adhesion ratio including borders (to phagocytosed)
update variable: adhC_RATIO_2 = ([adhC_NUM_1]\([adhC_NUM_1]+[phagC_NUM_1]))*100
Adhesion_aggregration
Aggregation ratio_adherent
update variable: on spores_map : agg_adh_RATIO = ([agg_adh_NUM]\[nonphagC_NUM_1])*100
Non-adhesion_aggregation
Aggregation ratio_non-adherent
update variable: on mphage_map : agg_nonadh_RATIO = ([agg_nonadh_NUM]\[nonphagC_NUM_1])*100
without borders' Mphages
Numbers
Number of all adherent conidia excluding borders' mphages, by position
compute statistical value: on spores_map conidia_adh with Number of linked objects Number of Linked via link_mphag-adhC_NB(all) within dist=999999, excl. current object > 0 at Spores_Level: adhC_NUM_2 = number
Number of all phagocytozed conidia excluding borders' mphages, by position
compute statistical value: on spores_map conidia_phag with Number of linked objects Number of Linked via link_mphage-phagC_NB(all) within dist=999999, excl. current object > 0 at Spores_Level: phagC_NUM_2 = number
Number of Mphages excluding borders
compute statistical value: on mphage_map mphages with Distance to scene border > 0 Pxl at Mphage_Level: mphage_NUM_2 = number
Number of macrophages having no associated conidia (excluding border's macrophages)
compute statistical value: on mphage_map mphages with Number of linked objects Number of Linked via link_mphage_allC_NB(all) within dist=999999, excl. current object = 0 at Mphage_Level: mphage_NUM_5 = number
Number of macrophages -excluding borders- with at least one conidium associated
update variable: mphage_Num_6 = [mphage_NUM_2]-[mphage_NUM_5]
SUM (adh+phag)
update variable: associatedC_NB_NUM = [adhC_NUM_2]+[phagC_NUM_2]
Ratio
Phag ratio by position
update variable: phagC_RATIO_2 = ([phagC_NUM_2]\([phagC_NUM_2]+[adhC_NUM_2]))*100
Phagocytosis Index
compute statistical value: mphages with Number of linked objects Number of Linked via link_mphage-phagC_NB(all) within dist=999999, excl. current object > 0 at Mphage_Level: mphage_NUM_3 = number
update variable: phagC_per_M_DIST = [phagC_NUM_2]\[mphage_NUM_2]

update variable: uptake_percentage_phag = [mphage_NUM_3]\[mphage_NUM_2]
update variable: phag_INDEX = [phagC_per_M_DIST]*[uptake_percentage_phag]
Adhesion Index
compute statistical value: mphages with Number of linked objects Number of Linked via link_mphag-adhC_NB(all) within dist=999999, excl. current object > 0 at Mphage_Level: mphage_NUM_4 = number
update variable: adhConPerMphage_RATIO = [adhC_NUM_2]\[mphage_NUM_2]
update variable: uptake_percentage_adh = [mphage_NUM_4]\[mphage_NUM_2]
update variable: adh_INDEX = [uptake_percentage_adh]*[adhConPerMphage_RATIO]
Adh ratio_adhTOphag_only detected
update variable: adhC_RATIO_3 = ([adhC_NUM_2]\([adhC_NUM_2]+[phagC_NUM_2]))*100
phagocytosing mphages Ratio
update variable: on Active Map : mphage_RATIO_1 = ([mphage_NUM_3]\[mphage_NUM_2])*100
update variable: mphage_RATIO_2 = ([mphage_NUM_4]\[mphage_NUM_2])*100
update variable: mphage_RATIO_3 = ([mphage_Num_6]\[mphage_NUM_2])*100
update variable: mphage_RATIO_4 = ([mphage_NUM_5]\[mphage_NUM_2])*100
Non-adherent Conidia_notknown
compute statistical value: on spores_map nonadhC_notknown at Spores_Level: notknownC_NUM = number
update variable: MOI = [totalC_seg_NUM]\[mphage_NUM_1]
update variable: index2 = [phagC_RATIO_2]*[mphage_RATIO_1]
My Export
assign class: on mphage_map mphages with Distance to scene border = 0 Pxl at Mphage_Level: _Temp1
export object statistics: on mphage_map mphages with Distance to scene border > 0 Pxl at Mphage_Level: export object statistics
export object statistics: on mphage_map conidia_adh, conidia_nonadh, conidia_phag at Mphage_Level: export object statistics
export object statistics: on mphage_map conidia_adh, conidia_nonadh, conidia_phag with Distance to scene border > 0 Pxl at Mphage_Level: export object statistics
export project statistics: on mphage_map : export project statistics
export project statistics: on mphage_map : export project statistics
export project statistics: on mphage_map : export project statistics
export project statistics: on mphage_map : export project statistics
export project statistics: on mphage_map : export project statistics
export project statistics: on mphage_map : export project statistics
export project statistics: on main : export project statistics
export project statistics: on main : export project statistics

export project statistics: on main : export project statistics
assign class: _Temp1 at Mphage_Level: mphages
Visualization_synchronizing both maps to mphage map
synchronize map: on spores_map conidia_adh, conidia_nonadh, conidia_phag, nonadhC_notknown at Spores_Level: synchronize map 'mphage_map'
display map: on mphage_map : display map 'mphage_map'
convert image objects: on mphage_map mphages, conidia_phag at Mphage_Level: convert image objects -> Connected 2D
merge region: background at Mphage_Level: merge region
